# Psychoneurological symptom clusters in patients with first stroke and relationship with inflammatory markers: a latent profile analysis

**DOI:** 10.3389/fpsyg.2025.1618513

**Published:** 2025-07-10

**Authors:** Yuanyuan Tang, Gudi Jiang, Meilan Jin, Shanyu Wu, Jinji Wu

**Affiliations:** ^1^School of Nursing, Yanbian University, Yanji, China; ^2^The Second Affiliated Hospital of Xuzhou Medical University, Xuzhou, China; ^3^Department of Neurology, Yanbian University Hospital, Yanji, China

**Keywords:** first stroke, psychoneurological symptom clusters, inflammatory markers, quality of life, a latent profile analysis

## Abstract

**Objective:**

This study aimed to explore the relationship between psychoneurological symptom clusters and inflammatory markers in patients with a first stroke.

**Methods:**

This study investigated 227 patients with a first stroke using general information questionnaires, Numerical Rating Scale, Pittsburgh Sleep Quality Index, Hospital Anxiety and Depression Scale, Ascertain Dementia 8, Fatigue Severity Scale, and Stroke-Specific Quality of Life. Data analysis included latent profile analysis, one-way analysis of variance, and unordered multicategorical logistic analysis.

**Results:**

The psychoneurological symptom clusters of first-stroke patients were categorized into three latent profiles: the low symptom group (45.2%), moderate symptom group (36.7%), and high symptom group (18.1%). Logistic regression analyses showed that younger age, female, higher National Institute of Health Stroke Scale score, higher interleukin-6 level, and higher hypersensitive C-reactive protein level were major predictive factors for the moderate and high symptom groups.

**Conclusion:**

Three latent profiles of psychoneurological symptom clusters exist in patients with a first stroke and are associated with markers of inflammation (interleukin-6 and hypersensitive C-reactive protein), thereby affecting their quality of life. These findings extend previous research on psychoneurological symptom clusters in stroke. Further exploration of a broader range of inflammatory markers and psychoneurological symptom clusters is necessary to advance symptom management.

## Introduction

Stroke is defined as an acute attack of neurological deficit caused by cerebrovascular complications, with corresponding clinical symptoms and signs. It is the second most common cause of death worldwide and the leading cause of disability (Niizuma et al., 2018). Patients diagnosed with stroke for the first time have high mortality and disability rates within 1 year (Tessua et al., 2021) and are susceptible to severe adverse psychological reactions and negative emotions that affect prognosis (Tan et al., 2023). Several studies have shown that patients with a first stroke usually experience symptoms such as anxiety, depression, fatigue, pain, sleep disturbance, and cognitive dysfunction concurrently, and there is a synergistic reinforcing effect between them, such that they form a cluster of psychoneurological symptoms, which exacerbates the patients’ symptomatic distress and seriously affects their quality of life (Katzan et al., 2019; Dong et al., 2022).

With the development of precision medicine and the rapid development of biobehavioral science research represented by omics, the National Institute of Nursing Research (NINR) revised the concept of symptom Science Model 2.0 in 2022 in response to the complexity of symptoms and the multiple correlations of their effects, adding three influencing factors: patient-centered experience, social determinants of health, and policy and population health. The integration of biobehavioral components, including biomarkers that influence psychoneurological symptom clusters, is designed to advance nurses’ understanding of the underlying biological mechanisms and biomarkers of symptoms and to develop targeted interventions to advance symptom management (Kurnat-Thoma et al., 2022). At present, there are few studies on psychoneurological symptom clusters and inflammatory markers, focusing on cancers such as breast (Al-Bashaireh et al., 2021) and prostate cancer (Sass et al., 2021).

Recent evidence has shown that because psychoneurological symptom clusters often coexist, some scholars have proposed that symptoms within these clusters may have the same pathogenesis and may be related to inflammatory markers (Kim and Malone, 2019). Lymphocytes and neutrophils are the most common peripheral blood markers of systemic inflammation, and sustained inflammatory response can lead to impaired cognitive function (Dhanesha et al., 2023), while neutrophil-to-lymphocyte ratio (NLR) can reflect the level of central nervous system inflammation to a certain extent and is related to mood and cognition (Buonacera et al., 2022). Interleukin-6 (IL-6) is significantly increased when the central nervous system is damaged and infected and is involved in immune regulation and inflammatory responses. This systemic inflammatory response can induce a range of disease behaviors such as anxiety, depression, and cognitive dysfunction (Cui et al., 2022). Studies have shown that an increased IL-6 concentration in the peripheral blood is associated with cognitive dysfunction, depression, and fatigue in the acute stage of stroke (Zhang et al., 2024). Hypersensitive C-reactive protein (hs-CRP) is strongly associated with nerve damage as an inflammatory agent, and high hs-CRP levels on admission are associated with sleep disturbance and fatigue. Miller et al. (2008) believe that fatigue, cognitive dysfunction, anxiety, and depression have the same neuroendocrine-immune pathophysiological mechanism, and inflammation may be their common underlying cause; that is, these conditions are related to inflammatory factors that are activated and released during inflammation and play a crucial role in psychoneurological symptom clusters. Although psychoneurological symptom clusters are associated with inflammatory markers, the specific mechanisms of action remain unclear. Therefore, exploring the association between stroke psychoneurological symptom clusters and inflammatory markers is necessary and could lead to the identification of new therapeutic targets aimed at alleviating symptoms and improving patient outcomes.

Latent profile analysis (LPA) focuses on individuals, classifies samples according to different characteristics or variables, classifies individuals into different categories, analyzes them at the individual level, and explains the correlation between external continuous variables using latent category variables. It is widely used in medicine, psychology, and other fields (Wang Y. et al., 2022). In this study, LPA was used to explore the potential categories of the psychoneurological symptom clusters of the first stroke; to deeply explore the relationship between the internal characteristics of the psychoneurological symptom clusters, inflammatory markers, and quality of life; to analyze the predictors of patients with severe symptoms; and to provide a basis for more targeted interventions for patients with severe symptom burden. Improving the efficiency of symptom management and quality of life of patients is of great significance.

## Methods

### Study design

Convenience sampling was used to select 227 patients with their first stroke who were hospitalized in the Department of Neurology of a tertiary-level hospital from January 2024 to May 2024 as the study subjects. This study was approved by the hospital’s ethical review board.

### Sample and setting

The baseline evaluation was conducted using an on-site questionnaire. Inclusion criteria: (a) confirmed by computed tomography or magnetic resonance imaging; (b) having a first stroke and in stable condition; (c) ≥ 18 years old; (d) being conscious and without hearing impairment; and (d) participating voluntarily and being willing to sign an informed consent form. Exclusion criteria were as follows: (a) patients with psychiatric disorders; (b) patients who were participating in other clinical studies and received psychological and psychiatric treatments; and (c) patients with a combination of other serious life-threatening diseases.

## Measurements

### Inflammation markers

Inflammatory markers (hs-CRP, IL-6) were measured from intravenous blood samples collected by the study nurses and stored at −80°C. Double-antibody sandwich enzyme-linked immunosorbent assay (ELISA) was performed according to the manufacturer’s protocol, and blood samples were tested in the same laboratory using the same equipment. The specimens were evaluated repeatedly, and the final results were averaged. The reliability of the inter- and intra-variability was < 10%. Inflammation Markers: + − Blinding: Laboratory technicians processing hs-CRP/IL-6 were blinded to patient grouping (double-blinded design), + − Batch Control:** All samples analyzed in duplicate across three batches with internal controls (CV < 8%), + − Interviewer Bias Mitigation: Scale administrators were blinded to biomarker results.

### Baseline information

The researchers collected sociodemographics including sex, age, marriage status, educational attainment, personality, place of residence, and total number of combined chronic diseases. Clinical characteristics including the National Institute of Health Stroke Scale (NIHSS) score and body mass index (BMI) were obtained from the patient’s electronic medical record.

### Numerical rating scale

The Numerical Rating Scale (NRS) was compiled by Turk et al. (1993) to assess the intensity of pain in patients over the past 24 h. A score of 0–10 indicates the degree of pain, with higher scores representing more severe pain. A score of 0 indicates no pain, 1–3 indicates mild pain, 4–6 indicates moderate pain, 7–9 indicates severe pain, and 10 indicates maximum pain. The NRS has been proven to be a valid and reliable method for measuring pain intensity.

### Pittsburgh Sleep Quality Index

The Pittsburgh Sleep Quality Index (PSQI) was compiled by Buysse et al. (1989) and consists of seven dimensions: subjective sleep quality, time to fall asleep, sleep duration, sleep efficiency, cognitive dysfunction, hypnotic medication use, and daytime dysfunction. Each dimension is graded on a scale of 0–3 for a total score of 0–21, with higher scores indicating poorer sleep quality. The Cronbach’s *α* coefficient for this study was 0.918.

### Hospital Anxiety and Depression Scale

The Hospital Anxiety and Depression Scale (HADS) was developed by Snaith and Zigmond (1986) and consists of seven entries each for the anxiety subscale (HAD-A) and depression subscale (HAD-D), with each entry scored on a scale of 0 to 3 and the total score ranging from 0 to 21. A score of zero to seven was classified as having no anxiety/depression symptoms, eight to 10 as mild anxiety/depression symptoms, 11 to 14 as moderate anxiety/depression symptoms, and 15 to 21 as severe anxiety/depression symptoms. The Cronbach’s *α* coefficient for this study was 0.857.

### Ascertain Dementia 8

The Ascertain Dementia 8 (AD8) was compiled by Galvin et al. (2005) and is commonly used to assess early cognitive dysfunction and contains 8 entries, with a score ≥2 suggesting possible cognitive dysfunction and <2 suggesting normal cognitive function. The Cronbach’s *α* coefficient in this study was 0.770.

### Fatigue Severity Scale

The Fatigue Severity Scale (FSS) was compiled by Krupp et al. (1989) and contains 9 entries, with each entry scored on a 7-point scale. The items are summed and divided by 9 to obtain the fatigue severity score, with a total score ≥4 indicating fatigue and a total score <4 indicating non-fatigue. The Cronbach’s *α* coefficient for this study was 0.955.

### Stroke Specific Quality of Life

The Stroke Specific Quality of Life (SS-QOL) was compiled by Williams et al. (1999) and includes 49 entries in 12 dimensions, such as language, energy, family roles, mobility, and work, graded using a Likert 5-point scale with each entry assigned a value of 1–5 points. The total score ranges from 49 to 245; the higher the score, the better the patient’s quality of life. The Cronbach’s α coefficient for this study was 0.878.

### Data collection

#### Test administration protocol

- Setting: All assessments conducted in private consultation rooms at the neurology department (ambient noise <35 dB, temperature 23 ± 2°C).
- Administrators: Two trained research nurses (5 + years neurology experience).
- Procedure:
  Step 1: Baseline questionnaires administered within 48 h of admission.
  Step 2: Venous blood drawn by nurses between 7:00–9:00 AM after overnight fasting.
  Step 3: Neuropsychological scales administered in fixed order (PSQI → HADS AD8 → FSS).
- Quality Control: Administrators received standardized training (*κ* = 0.91 for inter-rater reliability).

### Data analysis

Mplus 8.7 was used for latent profile analysis analysis of patients’ psychoneurological symptom clusters, and the LPA model fitting metrics included: the Akaike information criterion (AIC), the Bayesian information criterion (BIC), and the sample-corrected Bayesian information criterion (aBIC); the Roe-Mondale-Reuben likelihood ratio test metrics (LMR) and the bootstrapped-based likelihood ratio test metrics (BLRT) were used to compare the fitting differences of potential category models; entropy (Entropy) took the value of 0 ~ 1, the closer to 1 the higher the classification accuracy. SPSS software (version 26.0) was used to analyze the data. Measurement information conforming to normal distribution was expressed as mean ± standard deviation and one-way analysis of variance was used for comparison among multiple groups; counting information was expressed as the number of cases and percentage (%), and χ^2^ test or Fisher’s exact probability method was used for comparison among multiple groups. Variables that were significant in the comparison among multiple groups were further analyzed by unordered multicategorical logistic regression analysis. Statistical tests were performed. The difference was considered statistically significant at *p* < 0.05, and the test level was *α* = 0.05.

## Results

### Analysis and naming of potential profiles of psychoneurological symptom clusters in first-stroke patients

Four potential profile models were extracted in this study, and their model-fitting metrics are listed in Table 1. The absolute value of log (L) and the values of the AIC, BIC, and aBIC model fitting metrics decreased gradually with an increase in the number of categories. When the model was set to four latent profiles, the coefficients of the fit indicators AIC, BIC, and aBIC were the smallest; however, the *p* value of the LMRT indicator was not significantly different under the 4-category model. In addition, the values of the fitted metrics AIC, BIC, and aBIC were smaller under the 3-category model than under the 2-category model, and compared with the 2-category model, the 3-category model was able to distinguish the subcategories of the psychoneurological symptom clusters of first-stroke patients more finely. Therefore, considering the theoretical and practical significance of the fitting results and classification, the three categories were selected as the optimal models in this study based on previous studies.

**Table 1:** Indicators of model fit for potential categories of psychoneurological symptom clusters in first-stroke patients (*n* = 227).

Model	AIC	Log(L)	BIC	aBIC	Entropy	LMRT	categorical probability
1	6948.623	−3462.312	6989.723	6951.691	—	—	—
2	6200.077	−3081.038	6265.151	6204.935	0.994	<0.001	0.819/0.181
3	5907.866	−2927.933	5996.915	5914.514	0.924	<0.001	0.454/0.366/0.180
4	5843.819	−2888.910	5956.843	5852.256	0.910	0.248	0.419/0.079/0.322/0.181

AIC, The Akaike information criterion; BIC, Bayesian information criterion; LMRT, Lo–Mendell–Rubin adjusted likelihood ratio test.

**Figure 1:**
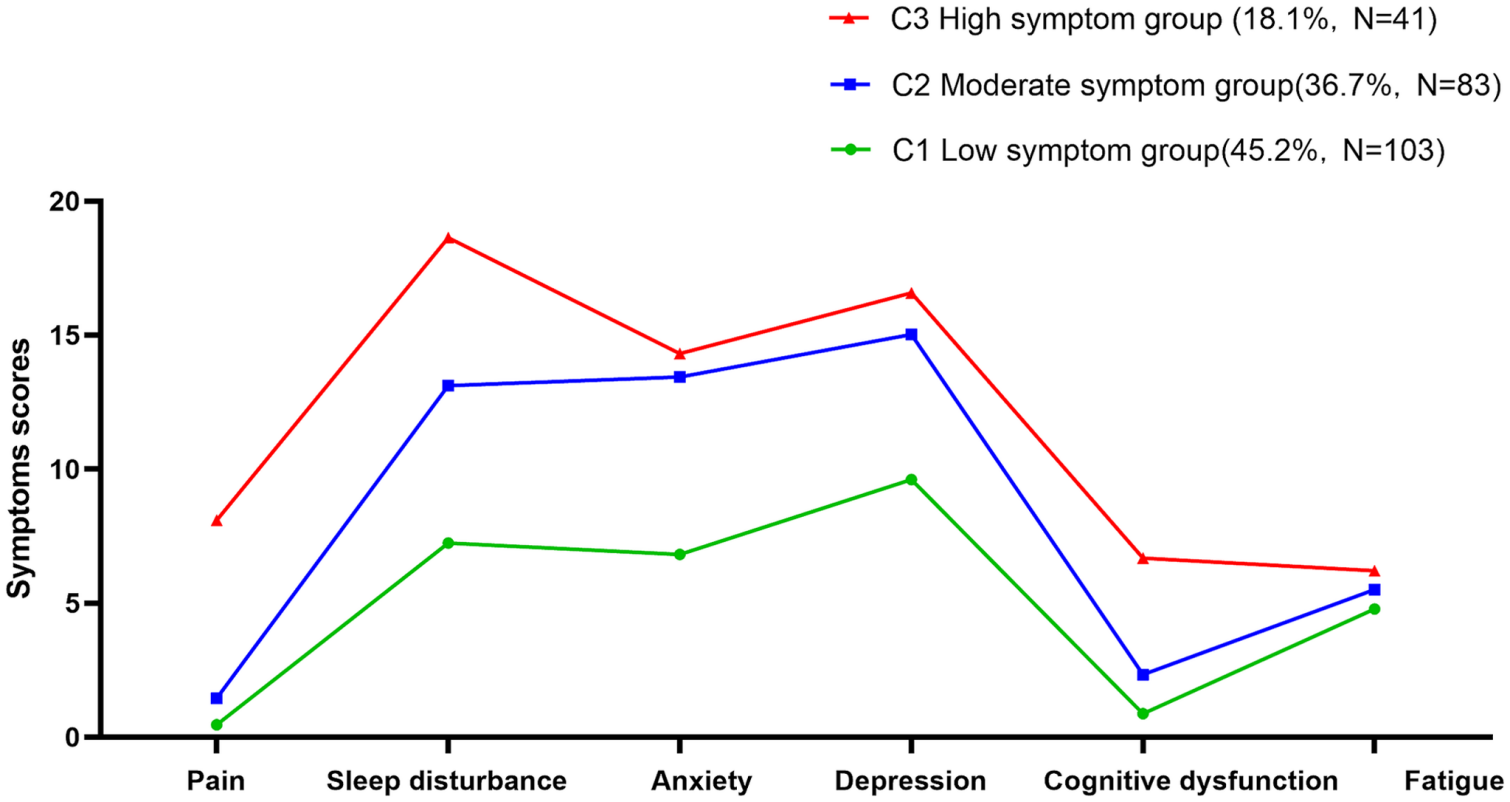
Potential profiling of psychoneurological symptom clusters in the first stroke patients.

As shown in Figure 1, the three potential category groups of psychoneurological symptom clusters in patients experiencing their first stroke showed different scores for pain, sleep disturbance, anxiety, depression, cognitive dysfunction, and fatigue. This study categorizes them into three categories, C1, C2, and C3. In category C1, patients had low scores on pain, sleep disturbance, anxiety, depression, cognitive dysfunction, and fatigue symptoms, so it was named the low symptom group with a ratio of 45.2%. In category C2, patients scored moderately well on pain, sleep disturbance, anxiety, depression, cognitive dysfunction, and fatigue symptoms, so it was named the moderate symptom group with a ratio of 36.7%. In category C3, patients pain, sleep disturbance, anxiety, depression, cognitive dysfunction, and fatigue symptoms were more severe than those in the other categories, so it was named the high symptom group, accounting for 18.1%.

### Differences in sociodemographic, clinical characteristics, inflammation markers, and total quality of life score across latent profiles

The differences among the three patient groups revealed that females predominated in both the low (63.1%) and high (61.0%) symptom categories, while males constituted the majority of moderate-symptom cases (65.1%) [χ^2^ = 16.088, *p* < 0.001]. Additionally, age [χ^2^ = 16.993, *p* < 0.001], personality [χ^2^ = 10.318, *p* = 0.035], place of residence [χ^2^ = 10.892, *p* = 0.004], and NIHSS score [χ^2^ = 30.005, *p* < 0.001] were statistically significant (*p* < 0.05). The total quality of life scores of patients in the low and moderate symptom groups were higher than those of patients in the high symptom group (*p* < 0.001). The hs-CRP level [*F* = 4.389, *p = 0.013*] was higher in patients in the high symptom group than that in the moderate symptom and low symptom groups, and the IL-6 [*F* = 5.629, *p* = 0.004] expression level was lower than that in the moderate and high symptom groups, whereas there was no statistically significant difference between the three groups in terms of neutrophil counts, lymphocyte counts, and neutrophil-to-lymphocyte ratios (*p* > 0.05). See Table 2.

**Table 2:** Differences in sociodemographic, clinical characteristics, inflammation markers, and total quality of life score across latent profiles (*n* = 227).

Variables	C1^①^ (n = 103)	C2^②^ (n = 83)	C3^③^ (n = 41)	χ^2^ or F	*P*
Sex					
Male	38(36.9)^a^	54(65.1)^b^	16 (39.0)^a^	16.088^c^	<0.001
Female	65(63.1)^a^	29(34.9)^b^	25 (61.0)^a^		
Age (years)					
<60	32(31.1)^a^	23(27.7)^a^	26(63.4)^b^	16.993^c^	<0.001
≥60	71(68.9)^a^	60(72.3)^a^	15(36.6)^b^		
Marriage status					
Spousal	91(88.3)	71(85.5)	32(78.0)	2.505^c^	0.286
Spouseless	12(11.7)	12(14.5)	9(22.0)		
Educational attainment					
Elementary school and below	14(13.6)	19(22.9)	11(26.8)	8.024^c^	0.236
Junior high school	37(35.9)	23(27.7)	17(41.5)		
Senior high school	26(25.2)	18(21.7)	6(14.6)		
College and above	26(25.2)	23(27.7)	7(17.1)		
Personality					
Introverted	40(38.8)^a^	45(54.2)^a,b^	26(63.4)^b^	10.318^c^	0.035
Intermediate	38(36.9)^a^	25(30.1)^a^	12(29.3)^b^		
Export-oriented	25(24.3)^a^	13 (15.7)^a^	3 (7.3)^b^		
Place of residence					
Municipalities	78(75.7)^a^	61(73.5)^a^	20(48.8)^b^	10.892^c^	0.004
Countryside	25(24.3)^a^	22(26.5)^a^	21(51.2)^b^		
Total number of combined chronic diseases					
0	23(22.3)	21(25.3)	9(22.0)	3.594^c^	0.464
1–2	79(76.7)	59(71.1)	32(78.0)		
≥3	1 (1.0)	3 (3.6)	0(0.0)		
NHISS score					
<4	91(88.3)^a^	66(79.5)^a^	19(46.3)^b^	30.005^c^	<0.001
≥4	12(11.7)^a^	17(20.5)^a^	22(53.7)^b^		
BMI (kg/m^2^)					
<18.5	6(5.8)	9(10.8)	4(9.8)	9.272^c^	0.159
18.5~	34(33.0)	32(38.6)	19(46.3)		
24.0~	43(41.7)	27(32.5)	16(39.0)		
28.0~	20(19.4)	15(18.1)	2(4.9)		
hs-CRP/(mg/L)	4.48 ± 2.65	4.82 ± 2.49	5.93 ± 2.97	4.389^d^	0.013
IL-6/(pg/mL)	4.78 ± 2.11	5.65 ± 2.56	6.24 ± 3.38	5.629^d^	0.004
Neu/(×10^9^/L)	3.95 ± 1.95	4.45 ± 1.63	4.56 ± 1.82	2.489^d^	0.085
Lym/(×10^9^/L)	1.47 ± 0.67	1.54 ± 0.70	1.71 ± 0.94	1.506^d^	0.224
NLR/(×10^9^/L)	3.8 ± 10.62	4.03 ± 3.86	4.49 ± 4.89	0.092^d^	0.912
SS-QOL total score	169.36 ± 30.43	145.20 ± 28.47	100.88 ± 10.12	94.116^d^	<0.001

BMI, body mass index; hs-CRP, hypersensitive-C-reactive protein; IL-6, interleukin-6; Neu, neutrophil; Lym, lymphocyte; NLR, neutrophil-to-lymphocyte ratio; SS-QOL, Stroke-Specific Quality of Life Scale; NHISS score, National Institute of Health Stroke Scale score.χ^2^: Chi-square test for categorical variables; F: ANOVA for continuous variables.a,b,x^2^-Test multiple comparisons different superscripts denote significant differences between groups, *p* < 0.05. c, χ^2^-Test; d, ANOVA analysis; d, F-test multiple comparisons.

### Multicategorical logistic regression analysis of latent profiles of psychoneurological symptom clusters in first-stroke patients

Statistically significant differences in the one-way analysis of variance (sex, age, personality, place of residence, NIHSS score, hs-CRP and IL-6 levels) were used as independent variables, and the latent profiles of symptom clusters were used as dependent variables (with the low-symptom group as the reference) in an unordered multicategorical logistic regression analysis, the results of which are shown in Table 3. The results indicated that younger age [B = 1.607, 95% CI (1.966, 12.664)], those with female [B = 1.169, 95% CI (1.716, 6.039)], higher NIHSS score [B = −1.842, 95% CI (0.058, 0.433)], higher hs-CRP [B = 0.221, 95% CI (1.055, 1.474)] and higher IL-6 [B = 0.321, 95% CI (1.146, 1.657)] levels were more likely to be classified in the moderate and high symptom groups.

**Table 3:** Multicategorical logistic regression analysis of potential categories of psychoneurological symptom clusters in stroke.

Variables	B	SE	Wald χ^2^	*P*	OR	95% CI
C2						
IL-6	0.171	0.076	5.028	0.025	1.187	[1.022, 1.378]
Sex	1.169	0.321	13.275	<0.001	3.219	[1.716, 6.039]
C3						
hs-CRP	0.221	0.085	6.683	0.010	1.247	[1.055, 1.474]
IL-6	0.321	0.094	11.627	0.001	1.378	[1.146, 1.657]
Age	1.607	0.475	11.439	0.001	4.989	[1.966, 12.664]
NIHSS score	−1.842	0.513	12.908	<0.001	0.159	[0.058, 0.433]

SE, standard error; CI, confidence interval; NIHSS score, National Institute of Health Stroke Scale score; hs-CRP (hypersensitive C-reactive protein) and IL-6 (interleukin-6) were included as measured, and only statistically significant variables are listed in the table.

## Discussion

This study explored the internal characteristics of the psychoneurological symptom clusters in a patient experiencing their first stroke based on LPA, which were ultimately divided into low-, moderate-, and high-symptom groups based on the statistical classification results combined with practical implications. Patients in the low symptom group had pain and cognitive functioning at a lower level and were less bothered by their symptoms. Patients in the moderate symptom group had moderate levels of sleep disturbance, anxiety, and depression, possibly due to altered physiological states, such as abnormal activation of the hypothalamic–pituitary-adrenocortical (HPA) axis, neurotransmitter disorders, altered neuronal excitability, and imbalanced cytokine levels (Frankiensztajn et al., 2020). Patients in the highly symptomatic group had a higher symptom burden, with sleep disturbance, depression, and cognitive dysfunction at a higher level than those in the other two groups. This may be related to the high impact of the first sudden stroke and the residual sequelae of varying degrees of severity, which were characterized by limb motor dysfunction, language dysfunction, and cognitive decline. The results of the classification in this study were generally consistent with those of Doong et al. (2015) in patients with breast cancer, confirming the plausibility that the measured psychoneurological symptom clusters can be regarded as the same. In contrast, Dong et al. (2022) conducted an analysis on the potential categories of psychoneurological symptom clusters of acute stroke, which were divided into “all low-symptom group,” “high psychological disorder,” and “all high-symptom group.” It is worth noting that our study showed that the line charts of the three groups were generally higher in sleep disturbance, anxiety, depression, and fatigue. The differences between studies may be due to the relatively large number of patients aged > 60 years included in the present study. Compared with younger patients, patients over 60 years of age have less anxiety and depression and more sleep disturbance and fatigue (Kapoor et al., 2019), which needs to be further verified. Contrary to prior reports, our cohort showed uniformly elevated symptoms across all domains in the youngest group (Group 3). This suggests that first-stroke survivors under 60 may experience global symptom escalation, potentially due to acute psychosocial stressors (Khan et al., 2023).

Synergistic effects can exist in symptom clusters compared with single symptoms, exacerbating the patient’s symptom burden and severely affecting their quality of life. Quality of life is of great concern as a major patient-reported clinical outcome (Kim et al., 2021). The present study showed that patients in the moderate and high symptom groups had lower quality of life scores compared with those in the low symptom group, suggesting that patients with multisymptom distress have a poorer quality of life, a finding that is generally in line with the findings of patients with type 2 diabetes (Li et al., 2019). This may be related to the patient’s denial and skepticism about the first diagnosis of stroke, which psychologically causes great pressure, often with personality and psychological changes manifested as depressed mood, irritability, and feelings of aggression and suspicion. They are also accompanied by cognitive dysfunction, anxiety, loss of appetite, generalized fatigue, and headache, severely affecting the psychological and physiological aspects of the patient, ultimately leading to a lower quality of life. The higher prevalence of introversion in moderate/high symptom groups may reflect maladaptive coping strategies (e.g., social withdrawal) exacerbating inflammation (Ike et al., 2020). While this contrasts with historical ‘Type A’ cardiovascular risk models (characterized by achievement-driven extroversion), introversion-related social isolation could heighten neuroinflammation via HPA-axis dysregulation (Gądek-Michalska et al., 2017). Notably, the lack of educational differences argues against achievement-motivation as a primary driver. Future studies should explore whether younger high-symptom patients exhibit stronger denial-inflammation links. Therefore, medical professionals should recognize patients with high psychological disorders at an early stage and encourage them to add non-pharmacological treatments, such as acceptance and commitment therapy or Pilates training, together with active medication.

The results of this study showed that younger age, female, and higher NIHSS scores were more likely to be categorized into the moderate and high symptom groups. Younger females faced elevated risks for moderate/high symptoms, but older females comprised most low-symptom cases. Younger males showed bimodal distribution across severity groups. This may be related to the sudden loss of ability to work in younger patients with their first diagnosis of stroke, which affects their ability to provide for their families and careers. In addition, younger patients are in a transition of social and family roles, worrying about disease prognosis and fearing disease recurrence. Women, on the other hand, are more prone to mental health problems due to their susceptibility to various external factors and lack of coping skills, which is consistent with the findings of Aleksandra (Pavlovic et al., 2022). While a high NIHSS score is considered an independent risk factor, sudden onset stroke can cause neurological deficits; the higher the degree of neurological deficits, the worse the patient’s psychological acceptance (Chang et al., 2022). Therefore, healthcare professionals should pay more attention to the predictors of psychoneurological symptom clusters in patients with a first-time stroke, which can help implement individualized interventions according to the patient’s condition.

This study showed that patients with high levels of hs-CRP and IL-6 expression were more likely to enter the moderate or high symptom groups, suggesting that inflammatory markers play a role in the pathogenesis of psychoneurological symptom clusters, which is consistent with findings in head and neck cancer and glioma (Li et al., 2023). Based on the theoretical scientific model of symptoms, we found that biological behavioral factors play an important role. Specifically, hs-CRP can induce vascular endothelial cells to produce a variety of cytokines and inflammatory cell chemokines and increase inflammatory mediators in the central nervous system, thereby triggering cognitive dysfunction and psychological disorders (Milosevich et al., 2024). IL-6 can activate neutrophils and increase inflammation, is associated with depression and fatigue, alters sleep disturbances, and aggravates cognitive dysfunction (Ji et al., 2017). Existing data show that immediately after an acute stroke, the immunoinflammatory response is activated in the central and peripheral nervous systems, and the expression of pro-inflammatory cytokines (such as IL-6 and hs-CRP) is significantly increased. This change initiates or amplifies the inflammatory response, leading to dysfunction of the norepinephrine system and hyperactivity of the HPA axis (Bach et al., 2019), which triggers a cluster of psychoneurotic symptoms in stroke patients. This further confirms that psychoneurological symptom clusters do not exist independently and that the higher the level of inflammatory cytokines, the more severe the symptoms.

In clinical practice, in addition to the use of non-steroidal anti-inflammatory drugs and other drug interventions to reduce psychological and neurological symptoms, more non-drug interventions are being used. The common rationale for these interventions is their potential to reduce inflammation, thereby alleviating symptoms and stroke recurrence risk. For example, mindfulness therapy and aerobic exercise suppress IL-6/hs-CRP production, while ear acupressure may modulate neuro-immune pathways. By targeting inflammation—a key mechanism identified in our study—these interventions address the biological basis of symptom clusters. Cognitive behavioral therapy can significantly improve the cognitive dysfunction-fatigue-anxiety-depression symptom cluster in patients with insomnia (Sweetman et al., 2021). Mindfulness therapy significantly improved symptoms of anxiety, depression, pain, and insomnia in patients with Parkinson’s disease (Bogosian et al., 2022). Studies have shown that acceptance and commitment therapies can effectively improve pain, anxiety, depression, and cognitive dysfunction in patients with acute cerebral infarction (Wang X. et al., 2022). In addition, moderate exercise relieves psychological symptoms and improves overall health by promoting endorphin secretion and reducing sympathetic nervous system activity. Practicing yoga can improve depression and fatigue symptoms and improve the quality of life of patients with breast cancer (Greaney et al., 2022). Moderate-intensity aerobic exercise has also been shown to reduce pain, anxiety, depression, and insomnia symptoms (Manojlović and Kopše, 2023). Recent evidence suggests that the Mediterranean diet can reduce depression and anxiety in people with multiple sclerosis and improve sleep quality. Traditional Chinese medicine can improve physical and mental health by regulating meridians and “qi” and “blood.” Studies have shown that ear acupoint compression can significantly reduce pain, fatigue, and sleep disturbances in patients with breast cancer (Yeh et al., 2016). Aromatherapy massage could reduce pain and cognitive dysfunction in patients with rheumatoid arthritis in the short term (Lu et al., 2023). Network analysis can also help to develop more effective symptom interventions by revealing the interactions between symptoms (Airaksinen et al., 2020). Therefore, medical staff should pay attention to the combined effect of drug and non-drug interventions to develop targeted measures to alleviate psychoneurological symptom clusters in stroke patients and ultimately improve their quality of life. The common rationale for these interventions is their potential to reduce inflammation, thereby alleviating symptoms and stroke recurrence risk. For instance, mindfulness therapy and aerobic exercise suppress IL-6/hs-CRP production, directly addressing the biological mechanism identified in our study.

## Limitations

This study had some limitations. First, the study was cross-sectional, the sample was limited to the scope of the research, and the sample size was small, which may have led to unstable results for potential classifications and limited the generalizability of the findings. In addition, the examination of anxiety, depression, and cognitive dysfunction relied on scales rather than professional diagnosis, and the collection of inflammatory markers was limited. Therefore, in the future, a large sample, multiple regions, and inclusion of more inflammatory markers should be included, and longitudinal studies should be conducted to explore the inflammatory mechanisms of psychoneurological symptom clusters in first-stroke patients.

## Conclusion

In summary, this study explored the association between psychoneurological symptom clusters and inflammatory markers through latent profile analysis and initially confirmed that the inflammatory factors IL-6 and hs-CRP play a role in psychoneurological symptom clusters in patients experiencing their first stroke. In the future, longitudinal studies can be used to further explore the trajectory of inflammatory markers and psychoneurological symptom clusters in stroke and to derive periods of high prevalence of psychoneurological symptom clusters over time, which will be beneficial for healthcare professionals to implement preventive and targeted interventions at times of high prevalence in different disease stages, provide symptom management measures, and improve the quality of life of patients.

## Data Availability

The datasets presented in this article are not readily available because this project is under research and the data cannot be made public. Requests to access the datasets should be directed to Jinji Wu, jjwu@ybu.edu.cn.
